# Employing graphene acoustoelectric switch by dual surface acoustic wave transducers

**DOI:** 10.1038/s41598-019-44689-z

**Published:** 2019-06-03

**Authors:** Ching-Ping Lee, Yu-Peng Hong, Man-Ting Shen, Chiu-Chun Tang, D. C. Ling, Yung-Fu Chen, Cen-Shawn Wu, Jeng-Chung Chen

**Affiliations:** 1 Department of Physics, National Tsing Hua University, Hsinchu, 30013 Taiwan; 2 Department of Physics, Tamkang University, Tamsui Dist., New Taipei City, 25137 Taipei, Taiwan; 3 Department of Physics, National Central University, Jhongli, 32001 Taiwan; 4 Department of Physics, National Changhua University of Education, Changhua, 50007 Taiwan

**Keywords:** Nanoscale devices, Graphene

## Abstract

We implement a logic switch by using a graphene acoustoelectric transducer at room temperature. We operate two pairs of inter-digital transducers (IDTs) to launch surface acoustic waves (SAWs) on a LiNbO_3_ substrate and utilize graphene as a channel material to sustain acoustoelectric current *I*_*ae*_ induced by SAWs. By cooperatively tuning the input power on the IDTs, we can manipulate the propagation direction of *I*_*ae*_ such that the measured *I*_*ae*_ can be deliberately controlled to be positive, negative, or even zero. We define the zero-crossing *I*_*ae*_ as ${I}_{ae}^{off}$, and then demonstrate that *I*_*ae*_ can be switched with a ratio ${I}_{ae}^{on}/{I}_{ae}^{off}\, \sim \,{10}^{4}$ at a rate up to few tens kHz. Our device with an accessible operation scheme provides a means to convert incoming acoustic waves modulated by digitized data sequence onto electric signals with frequency band suitable for digital audio modulation. Consequently, it could potentially open a route for developing graphene-based logic devices in large-scale integration electronics.

## Introduction

Graphene –a two-dimensional (2D) sheet of carbon atoms arranged in a honeycomb lattice– exhibits various unique properties beneficial for post-silicon electronics^1,2^. Recent developments in graphene field-effect transistors (GFETs) suggest that graphene holds great promise in radio frequency (RF) applications^3–5^. For digital electronics, adopting new materials as a successor to Si must perform excellent switching capabilities with a low off-state dissipation power and a high on/off current ratio^6,7^. Nevertheless, graphene shows a serious hurdle for its applications in logic circuits^2^, because the pristine graphene does not possess an energy bandgap^1^. As a result, GFET cannot be turned off efficiently, leading to a low on/off current ratio typically less than 10^2^. Subsequently, the research efforts are geared toward two different directions: engineering graphene material to open a bandgap^8,9^, or exploiting layered 2D semiconductors with a naturally occurring bandgap, e.g. transition metal dichalcogenides (TMDs) and black phosphorus (BP)^7^.

In this work, we report a different approach to implementing graphene for logic devices by utilizing acoustoelectric effects. Here graphene is used as a channel material to convert surface acoustic wave (SAW) into acoustoelectric current *I*_*ae*_. We will show that *I*_*ae*_ induced by dual SAWs can be modulated by discretizing RF signals. In this regard, a graphene acoustoelectric transducer(GAET) can function as a logic switch. The switching performance is demonstrated by the successful generation and detection of the digital text carried by *I*_*ae*_ with a switching rate up to few tens kHz.

A surface acoustic wave is an acoustic wave traveling along the surface of the piezoelectric materials, with its displacement amplitude exponentially decaying into the material so that it is roughly confined within one wavelength beneath the surface^10^. SAW can be induced by distributed comb-like metallic structures, such as interdigital transducers (IDTs), deposited on the surface of the piezoelectric substrate. Triggered by the piezoelectric effect, the RF input signal at the transmitting IDT stimulates the SAW. For a typical SAW device, a second IDT is employed, served as a signal processing unit and a transducer, to convert the acoustic waves back into a RF signal. Nowadays, the SAW devices have been widely used in various RF signal processing techniques for telecommunications and sensors^11,12^.

The propagation of SAW is sensitively influenced by the local changes of the host medium, which causes the variations of the SAW velocity *v*_*s*_ and the SAW attenuation factor Γ. For example, SAWs can interact with two-dimensional electron gas (2DEG) placed nearby and the corresponding changes in both *v*_*s*_ and Γ have been used to probe the distinct electronic states of 2DEG^13–16^. In addition, the interaction between the SAW and the charge carriers of 2DEG can also induce a macroscopic direct current, acoustic current *I*_*ae*_, which is known as the acoustoelectric effect.

The acoustoelectric properties of graphene have been extensively studied^17–25^. Owning to the linear energy dispersion and gapless nature of graphene, electrons in graphene can absorb sound waves over a wide frequency range^26^ and in theory Γ is strikingly diminished as the Fermi level *E*_*F*_ is tuned across the charge neutral point (CNP)^17,20^. However, graphene does not possess piezoelectricity because of its central symmetric lattice structure, unlike to GaAs-2DEG. The major obstacles in studying and utilizing the acoustoelectric effects of graphene lie on how to generate SAW and maintain its propagation under the control of *E*_*F*_. Early experiments reveal that SAW of graphene can be excited by placing graphene either on or in close contact to a substrate with high piezoelectricity, e.g. LiNbO_3_ substrate^18^. By incorporating ion liquid gate and IDTs, *E*_*F*_ of graphene can be tuned across CNP, and *I*_*ae*_ exhibits an ambipolar effect- the sign of *I*_*ae*_ is reversed associated with the change of charge carriers from n- to p-type^22,23^. Furthermore, Γ of grapehene is extremely weak, approximately ~0.4 to 6.8 *m*^−1^ depending on the carrier density *n*_*s*_, which is three orders of magnitude smaller than that of GaAs 2DEG systems^23^. These fascinating properties make graphene an ideal material for various acoustoelectric devices, ranging from acoustic tweezers, branch switch, flip-chip devices etc.^22,27–29^.

A theoretical model to describe acoustodynamic effects in semiconductors was developed by G. Weinreich^30^. The acoustic current in a closed-circuit measurement (or voltage in an open-circuit measurement) is induced by a loss of wave energy associated with a proportional loss of SAW momentum, which is analogous to a force applying on the absorber (the charge carriers of graphene in this study). For a 2D system, we can assume that the acoustic current density *j*^*ae*^ is proportional to Γ with the coefficient Λ and flows along the direction of SAW propagation^15,16,31^:

1 $${j}_{i}^{ae}={\rm{\Lambda }}{I}_{i}{\rm{\Gamma }}/{v}_{s},$$

here *i*(=*x* or *y*) is the spatial index, ${I}_{x(y)}(={I}_{x(y)}^{0}\exp (-{\rm{\Gamma }}x(y))$ is the intensity of the SAW propagating along the *x*(*y*)-direction. It has been known that Λ can be described by^16^

2 $${\rm{\Lambda }}=\sigma /{n}_{s}e,$$

where *σ* is the DC conductivity of graphene. We assume that both Γ and Λ are spatially uniform because graphene is an isotropic material. Note that one may need to treat Γ and Λ in a tensor form when the SAW propagates on an anisotropic substrate or the carriers are in the presence of an external magnetic field^15,16,31^.

Because of the ambipolar effect of graphene, ${j}_{i}^{ae}$ through graphene in the electron- and hole-rich regimes flows in opposite direction and vanishes at charge neutral region due to cancellation^22,23^. Consequently, one can define a true zero-current state or an “off”-state at CNP although the channel is not completely closed. On the other hand, a fair on/off ratio ~20 has been reported by defining an on/off state away from CNP in our earlier study^23^. In principle, if the off-state is set exactly at CNP, one can get a much higher ratio (>10^7^). There are competitive advantages to utilize GAET for logic devices. For commercial SAW filters used in the RF front-end, the device requires sufficient high-power durability. In general the SAW device can withstand power levels ≥30 dBm, which is high enough to generate *I*_*ae*_ with a decent S/N ratio. Moreover, no quiescence power source is needed because GAET is activated by the energy of the RF input signals received by the IDT transceiver. Nevertheless, if one operated GAET like GFET that the RF signal is sent through the gate electrode^3,4^, the modulation speed of GAET would be too slow for practical applications^23^. It is mainly because the ionic liquid is adopted for the gate electrode in the present GAET design^22,23^. Note that the gate electrode made of conducting materials will severely damp the propagation of SAWs. This is the key bottleneck for GAET to be used for the logic devices.

## Design Concept and Device Details

**Figure 1:**
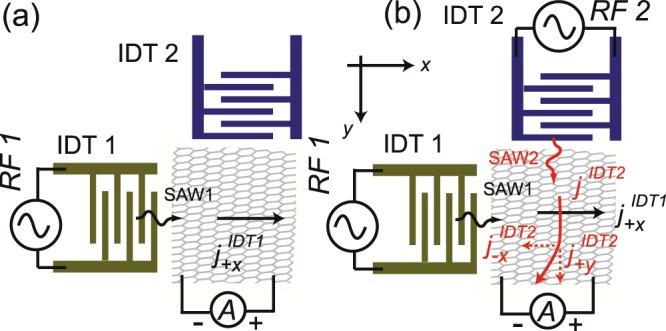
Schematics of the design concept for graphene acoustoelectric transducer (GAET). (**a**) The acoustoelectric current ${j}_{+x}^{IDT1}$ in graphene is generated by IDT1 and measured along the positive *x*-direction. (**b**) Both IDT1 and IDT2 are activated by two different RF signals. The measured acoustoelectric current is the sum of ${j}_{+x}^{IDT1}$ and ${j}_{-x}^{IDT2}$. Here we consider that graphene is in the hole-rich regime and its Fermi level is tuned away from the charge neutral point (CNP).

Our design concept is illustrated in Fig. 1(b). Two IDTs, denoted as IDT1 and IDT2, are employed on a LiNbO_3_ piezoelectric substrate in a nearly orthogonal arrangement. Each IDT comprises two sets of interleaved fingers and the acoustic current density induced by IDT1 and IDT2 is indicated as *j*^*IDT*1^ and *j*^*IDT*2^, respectively. Two current sensing leads are placed along the positive *x*-direction (cf. Fig. 1), and the measured acoustic current *I*_*ae*_ is determined by the vector sum of ${j}_{+x}^{IDT1}$ and ${j}_{-x}^{IDT2}$. The negative *x*-component of *j*^*IDT*2^ can be induced by deliberately adjusting orientation of IDTs or simply by the imperfection of the device. Therefore, we can manipulate the flow of *I*_*ae*_ by controlling the RF power separately applied on IDT1 and IDT2. As a result, the magnitude of *I*_*ae*_ measured could be positive, negative, or even zero. Our approach can be viewed as an application of acoustic-based active mixing technique, which has been widely used in studies of microfluidic channels with *I*_*ae*_ acting as the acoustic streaming of the sample liquid^32^. To make analogue to operation method of conventional field-effect transistor(FET), IDT1 functions as the source contact to inject the channel current and IDT2 serves as a gate electrode to turn “on/off” the device. We will demonstrate below that by digitizing RF signal applied on IDT2 or IDT1, the GAET can perform as a logic switch.

**Figure 2:**
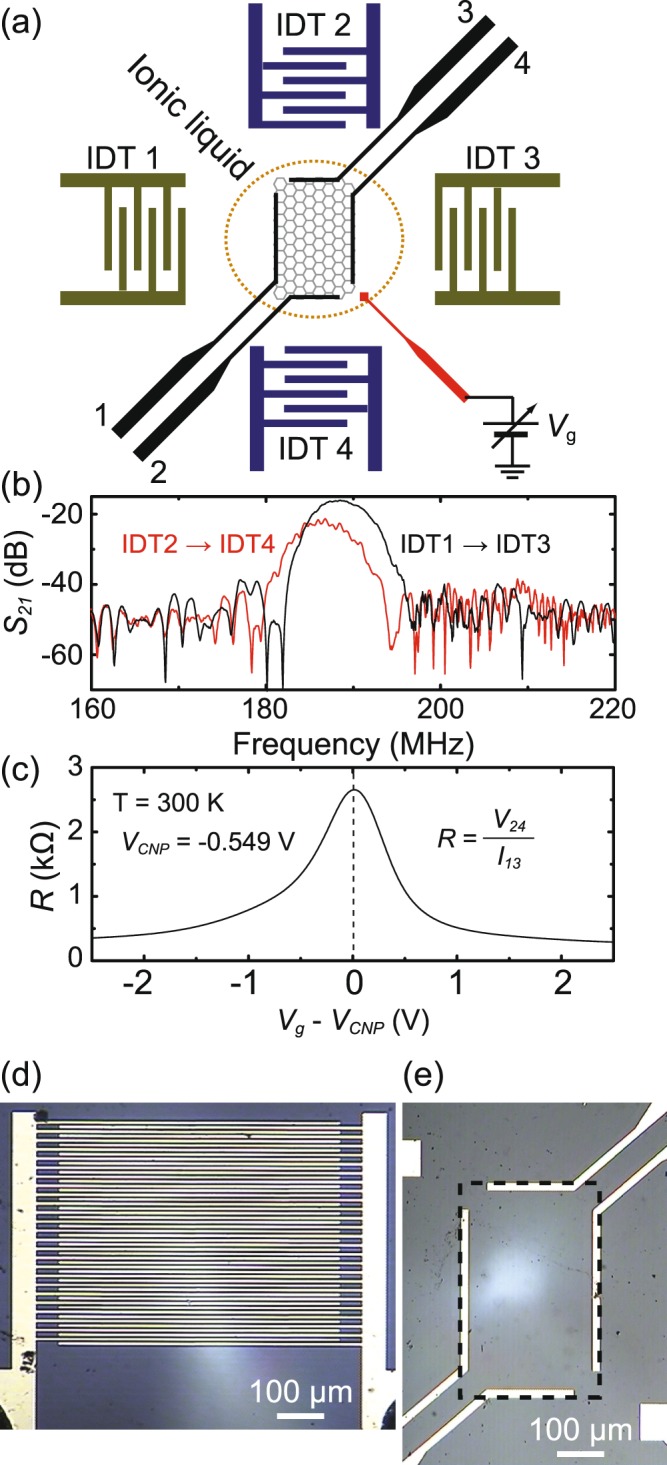
Device layout, characterizations and optical micrograph. (**a**) Schematic diagram of the studied GAET. (**b**) The transmission characteristics of SAWs taken with IDT1/IDT2 for launching SAW and IDT3/IDT4 for detection of SAW. It exhibits a peak at approximately 191 and 187 MHz, respectively. Here the input RF power is −10 dBm. (**c**) Graphene resistance *R* as a function of gate voltage *V*_*g*_ applied on the ionic liquid gate electrode. The resistance shows a maximum at the charge neutral point (CNP), where *V*_*g*_ = −0.549 V(≡*V*_*CNP*_). (**d**) The optical micrograph of IDT. Each IDT has 25 finger pairs made by 5 *μ*m wide electrodes. The length of overlapped electrode is 600 *μ*m. (**e**) The shape of graphene and the pattern of electrodes for graphene contacts. Graphene is tailored to a rectangular shape with 600 *μ*m in length and 400 *μ*m in width (frame with black dashed line). The longer electrodes are made with 450 *μ*m in length, and the shorter electrodes are made 250 *μ*m in length. All electrodes are made 20 *μ*m in width.

Figure 2(a) shows a schematic diagram of the investigated GAET. The device consists of two pairs of IDTs, denoted as IDT1 to IDT4, on LiNbO_3_ substrate, graphene, four electrodes on graphene labeled as leads 1 to 4, and a micro-bead of an ion-gel coated on graphene^33^, a gate electrode of the polymer electrolyte for applying gate voltage *V*_*g*_. The two sets of opposite ITDs, IDT1-IDT3 and IDT2-IDT4, are separated with a distance *L*_*T*_ = 1.4 mm and backed by metallic strips to damp reflected waves. In this study, we only operate IDT1 and IDT2, and conduct their counterpart IDT3 and IDT4 as a passive receiver for checking the SAW properties. Each IDT comprises two sets of interleaved fingers with *N*_*IDT*_ = 25 finger pairs made by 5 *μ*m wide electrodes with 8/70 nm of Cr/Au. The acoustic aperture *W*_*T*_ ~ 600 *μ*m, the overlap between electrodes, is aligned between two opposite IDTs along the [011] direction of the z-cut single-crystal LiNbO_3_ substrate. The optical micrograph of device can be found in the Fig. 2(d,e). The SAW wavelength *λ*_*SAW*_ determined by the pitch of the IDT electrode is 20 *μ*m and the SAW velocity *v*_*s*_ is approximately 3795 m/s^34^. The central resonance frequency *f* = *v*_*s*_/*λ*_*SAW*_ is estimated to be 190 MHz. Figure 2(b) shows the transmittance *S*_21_ as a function of frequency measured by a network analyzer (RS ZVA24). It exhibits a peak at approximately central frequency *f*_*c*_ = 191 and 187 MHz for IDT1 → IDT3 and IDT2 → IDT4 respectively, which fairly agrees with the designed value.

Graphene is prepared by the chemical vapor deposition. We refer the readers to our previous publications for the details of graphene growth, characterization, and transfer procedures^35–37^. Graphene is gently placed between two IDTs, and tailored to a rectangular shape of length *L*_*G*_ = 600 *μ*m and of width *W*_*G*_ = 400 *μ*m. Caution must be taken to ensure that graphene residues will not short the Au electrodes of IDTs. Four electrodes deposited along the side border of graphene are used for resistance and acoustoelectric current measurements. They are made of an Au/Cr bilayer with 8/70 nm in thickness and 20 *μ*m in width, among which 450 *μ*m in length for leads 1 and 4, and 250 *μ*m in length for leads 2 and 3 (see Fig. 2(a)). Finally, a micro-bead of the solid polymer electrolyte, poly ethylene oxide (PEO) and LiClO_4_^33^, is dropped onto graphene surface with size slightly larger than graphene area. Note that the geometry of the electrodes is designed in a way that the damping effect on SAWs due to intruding into the metallic electrodes is minimized and *I*_*ae*_ flowing along either longitudinal or transverse direction can be collected as much as possible. Figure 2(c) shows the representative resistance *R*(=*V*_24_/*I*_13_) of graphene as a function of *V*_*g*_, where *V*_24_ is the voltage measured across the lead 2 and 4, and *I*_13_ is the current passing through the lead 1 to 3. The *R* versus *V*_*g*_ trace of graphene reaches a maximum with resistance ~2.5 kΩ at CNP, where *V*_*g*_ ≡ *V*_*CNP*_ = −0.549 V. We have measured five devices with the same structures and got consistent results. Data presented below are mainly obtained from one of the devices.

## Results and Discussion

**Figure 3:**
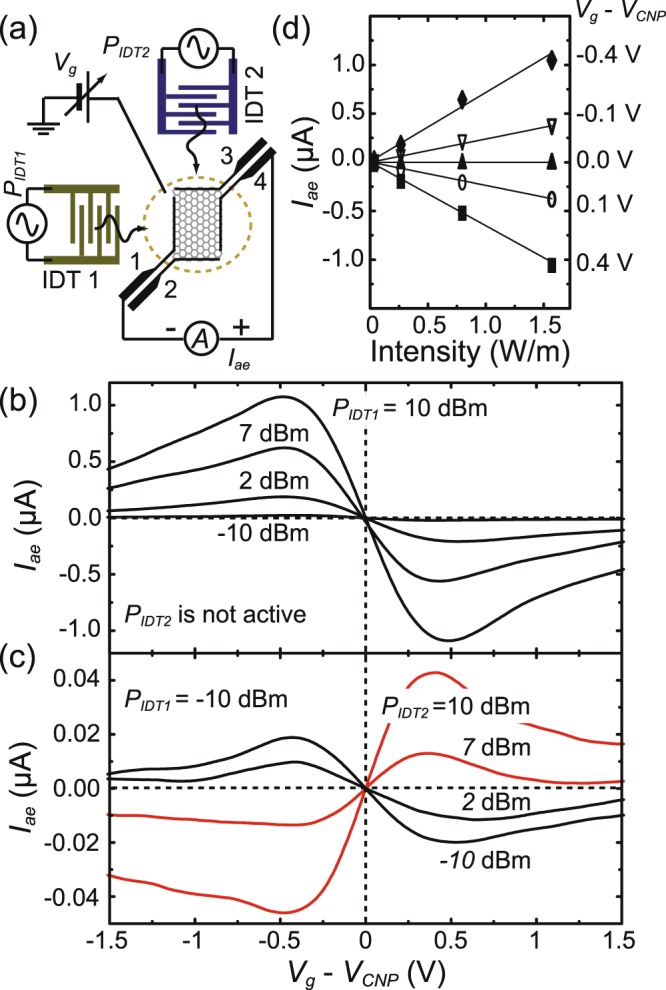
Acoustoelectric characteristics of the device. (**a**) The schematic plot of the *I*_*ae*_ measurement setup. (**b**) The quiescent performance of acoustoelectric current *I*_*ae*_ as a function of *V*_*g*_ at different RF-power *P*_*IDT*1_ applied on ITD1 at 191 MHz. Note that IDT2 remains unactivated. The majority carriers change from p- to n- type as the Fermi level is tuned across CNP, which causes a sign reversal of *I*_*ae*_. (**c**) *I*_*ae*_ versus *V*_*g*_ traces with various *P*_*IDT*2_ applied on IDT2. Here *P*_*IDT*1_ is kept at −10 dBm and both IDTs are operated at 191 MHz. When *P*_*IDT*2_ increases larger than 2 dBm, the polarity of measured *I*_*ae*_ changes. (**d**) Acoustoelectric current as a function of *P*_*IDT*1_ at various *V*_*g*_. Data are extracted from (**b**).

The acoustoelectric characteristics of the studied devices at room temperature is shown in Fig. 3. Figure 3(a) displays the experimental setup of *I*_*ae*_ measurement. Here we use IDT1 and IDT2 to generate the SAWs and take leads 4 and 1 to sense *I*_*ae*_, while keep the rest IDTs and electrodes inactive and open. We modulate RF signal at a frequency of 10 kHz and employ standard lock-in technique to measure *I*_*ae*_. Note that the propagation direction of the *I*_*ae*_ measured by leads 4 and 1 aligns with that of the SAWs induced by IDT1. Figure 3(b) shows *I*_*ae*_ as a function of bias voltage *V*_*g*_ with various RF powers *P*_*IDT*1_ applied on IDT1 at the central frequency of 191 MHz, while keeping IDT2 inactive. With the present arrangement of the current leads displayed in Fig. 3(a), the measured *I*_*ae*_ is positive, negative, or zero as graphene is biased in the hole-rich regime, electron-rich regime, or at *V*_*g*_ ~ *V*_*CNP*_. The gate bias dependence of *I*_*ae*_ manifests the unique Dirac dispersion relation of graphene^22,23^. We note that an on/off ratio of *I*_*ae*_ up to 10^7^ can be achieved, for example, if one defines the on-state at *V*_*g*_ − *V*_*CNP*_ = 0.5 V and the off-state at *V*_*g*_ = *V*_*CNP*_ for *P*_*IDT*1_ = 10 dBm. Extracted from Fig. 3(b), the measured acoustoelectric current as a function of the SAW intensity is plotted in Fig. 3(d). Within the applied RF power up to 10 dBm the acoustoelectric current is linearly proportional to the SAW intensity^18^, as indicated in Eq. (1).

To present the performance of the device in the dual-SAW operation, we cooperatively activate IDT1 and IDT2 at frequency of 190 MHz. First, we launch the SAWs by IDT1 with a fixed *P*_*IDT*1_ ~ −10 dBm to induce a steady positive *I*_*ae*_ on graphene in the hole-rich regime, and then gradually increase the input RF power *P*_*IDT*2_ on IDT2 ranging from −10 dBm to 10 dBm. Figure 3(c) shows *I*_*ae*_ as a function of applied bias *V*_*g*_ with various *P*_*IDT*2_. It is found that the value of *I*_*ae*_ decreases/increases with the increase of *P*_*IDT*2_ in the hole-/electron-rich regime. As *P*_*IDT*2_ > 2 dBm, *I*_*ae*_ almost diminishes. While *P*_*IDT*2_ increases further, *I*_*ae*_ remarkably changes sign, and its magnitude increases with *P*_*IDT*2_. The evolution of *I*_*ae*_ with *P*_*IDT*1_ and *P*_*IDT*2_ is inconsistent with the mixing-flow of *j*^*ae*^ scenario described in Fig. 1(b). However, the experimental findings demonstrate that the dual-SAW operation can null acoustoelecctric current in a controllable manner, which provides an alternative route to turn “off” *I*_*ae*_.

**Figure 4:**
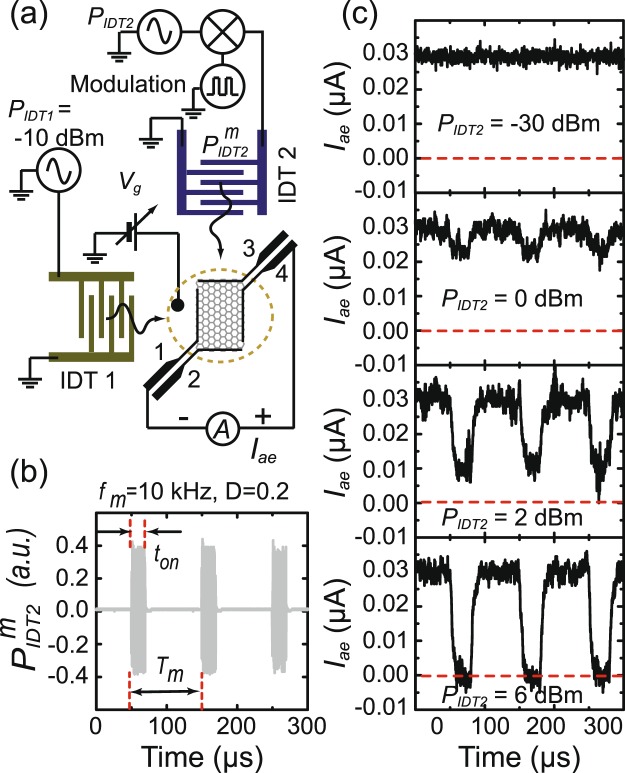
Time-resolved measurement of acoustoelectric current *I*_*ae*_. (**a**) Schematic diagram of the measurement circuit. Graphene is biased in the hole-rich regime at *V*_*g*_ − *V*_*CNP*_ = −0.5 V. (**b**) The time dependence of the amplitude modulated *P*_*IDT*2_ with a duty ratio *D*(≡*t*_*on*_/*T*_*m*_) = 0.2, where *T*_*m*_(=1/*f*_*m*_) is the modulation period. (**c**) The time dependence of *I*_*ae*_ with different modulated *P*_*IDT*2_ ranging from −30 dBm to 6 dBm. Here both IDTs is excited at 190 MHz. *P*_*IDT*1_ is fixed at ~−10 dBm, and *P*_*IDT*2_ is modulated with frequency *f*_*m*_ = 10 kHz.

Next we will show that by dynamically controlling the on/off state of *I*_*ae*_, GAET can be effectively operated as a logic switch. Figure 4(a) displays schematic diagram of the measurement circuit for real-time response measurements on *I*_*ae*_. We first bias graphene in the hole-rich regime at *V*_*g*_ − *V*_*CNP*_ = 0.5 V and then simultaneously apply a constant *P*_*IDT*1_ = −10 dBm on IDT1 and a modulated *P*_*IDT*2_ on IDT2 to generate a time-varying *I*_*ae*_. The open-circuit voltage *V*_*SAW*_ associated with the induced *I*_*ae*_ is amplified by a wide-band low noise amplifier and then is directly recorded through a digital oscilloscope with a bandwidth of 100 MHz and a sampling rate up to 1 GHz. The output voltage *V*_*SAW*_ corresponding to *I*_*ae*_ ~ 1 *μ*A is approximately 1 *μ*V. The RF-power *P*_*IDT*2_ is amplitude modulated by a square wave with a period *T*_*m*_(=1/*f*_*m*_ = 100 *μ*s) and a duty cycle *D*(=0.2). Oscilloscope traces of the applied RF-signal on IDT2 is shown in Fig. 4(b) for reference. Figure 4(c) shows the screen shot of the output waveforms of *I*_*ae*_ taken from the oscilloscope at various *P*_*IDT*2_. As *P*_*IDT*2_ is within active time of the pulse, *I*_*ae*_ exhibits a dip feature due to the cancellation by negative *I*_*ae*_. As *P*_*IDT*2_ increases up to 6 dBm, *I*_*ae*_ is nearly vanished. Here the GAET functions as an active-High logic switch that processes information as either a “1” or a “0”, depending on whether the switch is off − getting finite acoustoelectric current *I*_*ae*_− or on − *I*_*ae*_ measured zero. We estimate the on/off ratio of *I*_*ae*_ is approximately 10^4^ based on the noise level of the on and off states.

**Figure 5:**
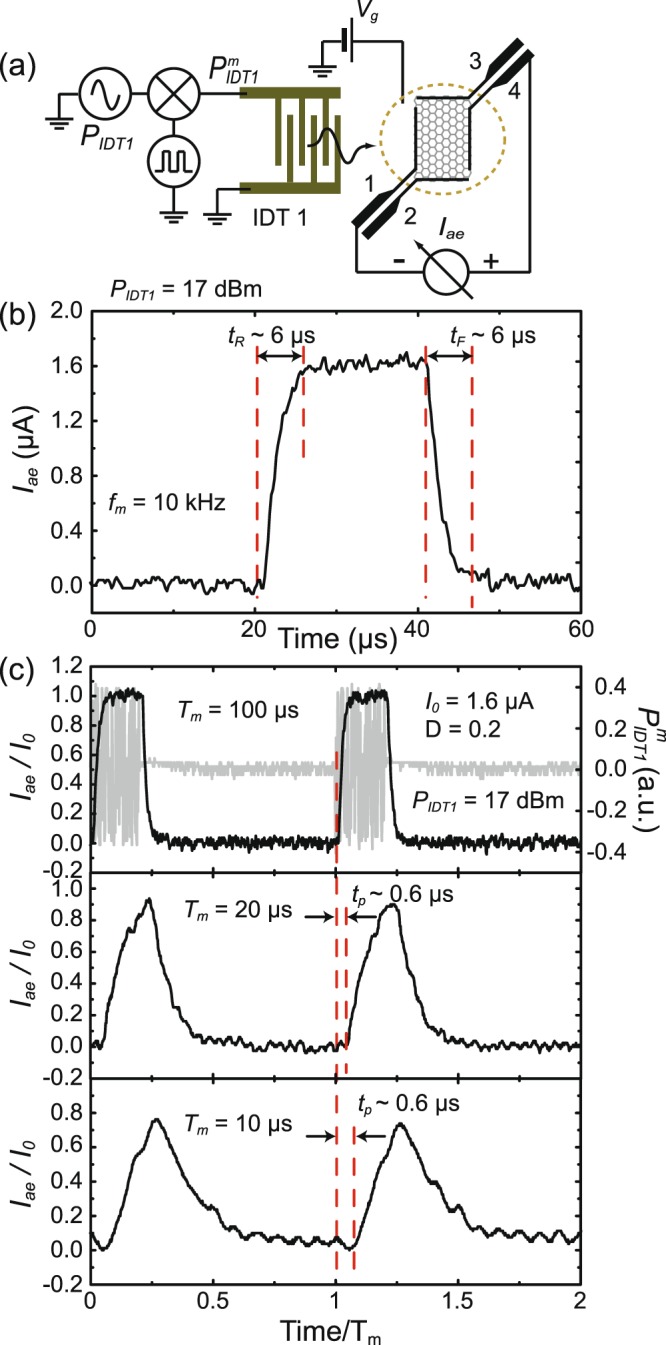
Characteristics of the transition rate of the graphene acoustoelectric switch. (**a**) Circuit diagram for the measurement. IDT1 is activated by a square wave-modulated RF-signal ${P}_{IDT1}^{m}$, where IDT2 is inactive. (**b**) A representative waveform of *I*_*ae*_ illustrates the step response of the device. Here *t*_*on*_ is set to be 20 *μ*s and the Rise *t*_*R*_ and Fall *t*_*F*_ time is found to be about 6 *μ*s. (**c**) The time dependence of *I*_*ae*_ with power modulation period of 100 *μ*s, 20 *μ*s, and 10 *μ*s. Here *P*_*IDT*1_ is fixed at 6 dBm and the duty cycle *D* = 0.2. The gray trace shown in the upper panel is the time trace of the modulated RF-signal ${P}_{IDT1}^{m}$ applied on IDT1. To compare how the transition response of *I*_*ae*_ digital pulse evolves with different *T*_*m*_, *I*_*ae*_ is normalized to its peak value *I*_0_(=1.6 *μ*A), and the time scale is normalized to *T*_*m*_.

To characterize the response time of switching *I*_*ae*_, we note that the maximum switching rate - the key parameter to limit sampling rate in digital communications - is determined by the transition time of *I*_*ae*_ in response to the modulated RF pulse. We can switch *I*_*ae*_ simply by modulating RF signal applied on a single IDT. Unlike the dual-SAW scheme discussed above, one can view such operation as an active-Low logic switch. In terms of GFET, graphene provides a nature 2D conducting channel such that a digital on/off state can be simply achieved by modulating the source-drain bias without applying a gate voltage, if the signal gain is not a concern. On the other hand, a RF signal can be directly converted to an electric signal in GAET. In this regard, GAET has an advantage over GFET as a logic switch. Figure 5(a) shows the circuit diagram to switch *I*_*ae*_ by operating IDT1 alone. Figure 5(b) shows the detailed profile of the *I*_*ae*_ pulse waveform generated by a square wave-modulated *P*_*IDT*1_(=17 dBm). The on-time *t*_*on*_ is set about 20 *μ*s. Based on the 90% and 10% threshold levels of the pulse amplitude, we determine the Rise *t*_*R*_ and Fall *t*_*F*_ time to be about ~6 *μ*s. Figure 5(c) shows the evolution of *I*_*ae*_ with different modulation frequencies. For comparison, we normalize *I*_*ae*_ to its quiescence value *I*_0_(=1.6 *μ*A), and time to the modulated period *T*_*m*_. As displayed in Fig. 5(c), the on-state remains stable with *T*_*m*_ up to 20 *μ*s, corresponding to dynamic switch rate of 50 kHz. That is to say, the peak value of the *I*_*ae*_ waveform with a pulse width ~4 *μ*s is within 90% of the full amplitude. The propagation delay time *t*_*p*_ - a parameter to evaluate the jitter effects- is about 0.6 *μ*s. We find ${t}_{p} \sim \ell /{v}_{s}$, where $\ell (\, \sim \,1.4\,{\rm{mm}})$ is the separation between IDT1 and the center of graphene. We estimate the digital modulation rate of ~10 KB/s for the GAET switch.

Data shown in Figs 4(c) and 5(c) present a way to switch on/off channel current by the digitized RF power without resorting the gate voltage, as long as graphene is intentionally doped away from the CNP. The liquid gate is not necessary for the GAET switch. On the other hand, we have affirmed that the switch rate is not affected by the presence of the ionic liquid nor the instrument. We estimate the capacitance of a single IDT *C*_*IDT*_ ~ 6.25 pF and the circuit input impedance around 448 Ω, giving rise to the RC time constant around 2.8 ns, which is much shorter than the *t*_*R*_ and *t*_*F*_ measured. Therefore, we argue that the intrinsic RC delay is likely due to the impedance mismatch. We conceive the switching rate can be immediately raised up by shrinking the channel width of graphene. To optimize impedance-matching one may employ tapered IDTs, functioning as an impedance-transformer^38–40^, which impedance match to 50 Ohm transmission line at one end and to the characteristic impedance of the tailored graphene and the leads at other end. To this end, one also needs to characterize the output impedance of GAET and match it with that of the transmission line extended to the measurement ports.

The dynamics of acoustoelectric effects of emerging post-graphene 2D materials – e.g. transition metal dichalcogenides (TMDs) and black phosphorus (BP) are much less explored^41^ and would be interesting subjects for future studies.

Finally, we wish to make few comments on future development of GAET logic devices. The ultimate response time of the GAET switch is limited by the SAW velocity *v*_*s*_ and the channel width. There are several approaches to increasing the switching rate. One may try to fabricate the device on substrates with a relatively large electromechanical coupling coefficient, e.g. 42° Y-X LiTaO_3_ or 64° Y-X LiTaO_3_ substrate, which have been widely applied to the SAW devices for mobile communications. However, the tradeoff is that larger leaky wave may yield a lower *I*_*ae*_. In principle, a high-slew rate of *I*_*ae*_ can be obtained from a wide-band SAW device, which can be implemented by an apodized IDT design or simply reducing the number of fingers in the IDT. A narrower channel width may give a shorter response time, but it in turn reduces *I*_*ae*_ or requires a larger *P*_*IDT*_. This drawback makes GAET unsuitable for latch operation. Regarding the operation scheme, a single IDT is sufficient for the active-Low switch. Using collinear dual IDTs such as IDT1 and IDT3 (or IDT2 and IDT4), one can apply lower and balanced *P*_*IDT*_ for the active-High switch. Nevertheless, evident interference due to reflected waves should be taken into account. For the dual-SAW operation, two SAWs can be excited at different frequencies. However, it makes SAW attenuation become more pronounced at a higher frequency. In addition, by properly utilizing four leads and IDTs, we can directly measure *I*_*ae*_ to make GAET act as an acoustoelectric branch switch^28^. We note that recent studies reveal several intriguing interface elastic properties of van der Waals materials^42–44^ and may offer a mean to speed up the switch rate of GAET by engineering the interfacial acoustoelectric properties. Maybe the switch rate is slow (audio frequencies) and the design is a little complex, we think that the GAET opens a route for developing graphene-based logic switch. In this work, we only demonstrate the feasibility of GAET as a logic switch and leave aforementioned issues for future studies.

## Conclusion

In conclusion, we present an accessible operation scheme of GAET for a logic switch with a moderate on/off rate of ~10^4^ at room temperature. By manipulating the propagation direction of *I*_*ae*_, the measured values of *I*_*ae*_ can be fine tuned to zero - an ideal off state for a logic switch. We demonstrate the dynamic switch rate of *I*_*ae*_ can be up to 50 kHz by modulating the amplitude of the input RF-signal applied on IDTs. By deliberately controlling the digitized RF-power applied on a pair of crossed-IDT or a single IDT, the output *I*_*ae*_ can be either active-High or active-Low, respectively. The digital modulation rate can achieve ~10 KB/s. The performance of the GAET is suitable for processing digital audio signals. Even the switch rate is slow, our work provides a means to integrate the SAW device and the acoustoelectric effects for future development of graphene-based logic devices.
